# Proteome-Wide Profiling of Lysine 2-Hydroxyisobutyrylation in *Vibrio alginolyticus* and Preliminary Association of CobQ with Global Khib Levels

**DOI:** 10.3390/life16101698

**Published:** 2026-10-10

**Authors:** Xiaoxin Wen, Shuai Yang, Yashu Liang, Yujia Zhang, Yingzhu Wei, Weijie Zhang, Jichang Jian, Na Wang, Bijian Pang, Huanying Pang

**Affiliations:** 1 Fisheries College, Guangdong Ocean University, Zhanjiang 524088, China; 2 Guangdong Provincial Key Laboratory of Aquatic Animal Disease Control and Healthy Culture & Key Laboratory of Control for Diseases of Aquatic Economic Animals of Guangdong Higher Education Institutes, Zhanjiang 524088, China; 3 Chinese Academy of Inspection and Quarantine, Beijing 100176, China; 4 Marine Environmental Monitoring Center of Guangxi, Beibu Gulf Marine Ecological Environment Observation and Research Station of Guangxi, Beihai 536000, China

**Keywords:** *Vibrio alginolyticus*, lysine 2-hydroxyisobutyrylation modification, CobQ, post-translational modification

## Abstract

*Vibrio alginolyticus* is an important opportunistic pathogen in marine aquaculture, causing vibriosis in a wide range of aquatic animals. Lysine 2-hydroxyisobutyrylation (Khib) is an emerging lysine acylation modification involved in regulating metabolism, stress responses, and pathogenicity in microorganisms. However, the global Khib modification profile and its potential biological functions in *V. alginolyticus* have remained completely unexplored. This study aims to characterize the Khib modification profile of *V. alginolyticus* and to preliminarily evaluate the role of CobQ as a candidate regulator of Khib homeostasis. A Khib-modified proteome of *V. alginolyticus* was constructed using affinity enrichment combined with LC-MS/MS-based modified proteomics. Bioinformatics analyses (GO, COG, KEGG, subcellular localization, motif, and protein–protein interaction) explored the functions of Khib-modified proteins. Selected proteins were analyzed by immunoprecipitation combined with Western blot. A *cobQ* deletion mutant strain was constructed to assess the association between *cobQ* deletion and global Khib levels, and to evaluate selected physiological traits. A total of 15,195 Khib sites were identified in 2848 proteins, indicating widespread distribution of Khib modification. Khib-modified proteins were mainly associated with metabolic processes, genetic information processing, signal transduction, membrane biogenesis, and secretion-related pathways. Motif analysis revealed conserved amino acid patterns surrounding Khib sites. Immunoprecipitation combined with Western blot confirmed the Khib modification of TpiA and Ndk at the protein level, but did not validate the specific LC-MS/MS sites. Deletion of *cobQ* was associated with increased global Khib levels, suggesting that CobQ may be associated with Khib modification in vivo. In vitro assays showed no statistically significant de-Khib activity of CobQ, although a trend toward lower Khib levels was observed. Phenotypic assays showed that *cobQ* deletion did not significantly affect growth or extracellular protease activity, but reduced swimming motility at low agar concentrations. This study provides a comprehensive catalog of putative Khib-modified proteins of *V. alginolyticus*. Our findings suggest that CobQ may be associated with global Khib levels, but its direct enzymatic activity and physiological substrates remain to be established. These findings provide a foundational resource for understanding Khib-mediated regulation in *V. alginolyticus* and identify candidate targets for future mechanistic studies.

## 1. Introduction

*Vibrio alginolyticus* is a halophilic and mesophilic Gram-negative bacterium widely distributed in marine environments and various marine animals [1]. At water temperatures of 25–35 °C, it causes severe vibriosis in economically important aquatic animals, including fish, shrimp, and shellfish [2]. In addition to its significance in aquaculture, *V. alginolyticus* is also recognized as an opportunistic zoonotic pathogen capable of causing food poisoning, otitis media, wound infections, and septicemia in humans. The pathogenicity of *V. alginolyticus* is associated with multiple virulence-related factors, including quorum sensing systems, adhesion factors, extracellular products, lipopolysaccharides, biofilms, iron acquisition systems, and secretion systems [3,4,5,6,7,8,9].

The virulence of *V. alginolyticus* is governed by a complex regulatory network. This network encompasses both effector mechanisms, such as the interference of extracellular products with host immunity, and coordination systems, such as the quorum sensing-mediated regulation of virulence factors. These processes require the precise expression and functional activation of numerous proteins. The activity, stability, and interaction patterns of these key proteins are, in turn, often fine-tuned by post-translational modifications (PTMs) [10,11].

Lysine 2-hydroxyisobutyrylation (Khib) is an emerging lysine acylation modification characterized by the covalent attachment of a 2-hydroxyisobutyryl group to the ε-amino group of lysine residues. Since its discovery, Khib has been shown to participate in the regulation of transcription, translation, protein degradation, metabolism, and stress responses [10]. In eukaryotes, several Khib regulatory enzymes have been reported, including Tip60 and p300 as writers and HDAC1, HDAC2, and HDAC3 as erasers [10,11]. In prokaryotes, TmcA has been identified as a lysine 2-hydroxyisobutyryltransferase, while CobB has been reported to possess de-2-hydroxyisobutyrylase activity in *Escherichia coli* [12,13]. These findings suggest that Khib may be a conserved and functionally important regulatory modification in both eukaryotic and prokaryotic organisms.

In *V. alginolyticus*, previous proteomic studies have revealed that other lysine acylation modifications, such as lysine acetylation and lysine succinylation, are widely distributed and may participate in the regulation of metabolism and virulence-associated traits [14,15]. However, the global distribution, functional characteristics, and potential regulatory proteins of Khib in *V. alginolyticus* remain unknown. Moreover, whether Khib modification is associated with physiological adaptation and virulence-related processes in this aquatic pathogen has not been systematically investigated.

In the present study, we performed the first proteome-wide analysis of Khib modification in *V. alginolyticus*. A total of 15,195 Khib sites were identified in 2848 proteins. Bioinformatics analyses were conducted to investigate the potential functions, enriched pathways, conserved motifs, and interaction networks of Khib-modified proteins. In addition, we investigated CobQ (Accession No: PQ351403) as a candidate regulator of global Khib levels. Although *cobQ* is annotated as a cobyric acid synthase, recent studies have shown that CobQ homologs in other bacteria possess lysine deacetylase activity, suggesting a potential role in regulating lysine acylation modifications. We therefore compared global Khib levels between the wild type and the *cobQ* deletion mutant, and evaluated selected physiological traits through genetic manipulation, Western blot analysis, and physiological assays. This study provides a comprehensive resource for understanding Khib modification in *V. alginolyticus* and offers a basis for further investigation of lysine acylation-mediated regulation in aquatic bacterial pathogens.

## 2. Materials and Methods

### 2.1. Bacterial Strains and Plasmids

The wild-type *V. alginolyticus* strain HY9901 was used in this study and maintained in our laboratory [16]. All strains and plasmids used in this study are listed in Table 1 and Table 2.

### 2.2. Protein Extraction and Khib Peptide Enrichment

*V. alginolyticus* HY9901 was cultured in 50 mL of TSB medium at 28 °C with shaking at 200 rpm. Cells were harvested after 24 h, washed three times with phosphate-buffered saline (PBS), and ground in liquid nitrogen. Three independent biological replicates were performed. Four volumes of lysis buffer containing 8 M urea, 1% protease inhibitor cocktail, 3 μM trichostatin A, and 50 mM nicotinamide were added to the samples, followed by sonication on ice for 3 min. After centrifugation at 12,000× *g* for 10 min at 4 °C, the supernatant was collected. Protein concentration was determined using a BCA assay kit (Thermo Fisher Scientific, Waltham, MA, USA). Approximately 5 mg of protein from each replicate was used for subsequent experiments. The proteins were precipitated with 20% trichloroacetic acid at 4 °C for 2 h.

The precipitated proteins were washed three times with pre-cooled acetone, air-dried, and redissolved in 200 mM triethylammonium bicarbonate. Trypsin (Promega, Madison, WI, USA) was added at a ratio of 1:50 enzyme to protein, and digestion was performed overnight at 37 °C. The peptides were reduced with 5 mM dithiothreitol at 56 °C for 30 min and alkylated with 11 mM iodoacetamide for 15 min in the dark. The peptide mixture was desalted using a Strata X solid-phase extraction column.

For Khib peptide enrichment, peptides were dissolved in NETN buffer containing 100 mM NaCl, 1 mM EDTA, 50 mM Tris-HCl, and 0.5% NP-40 at pH 8.0. The peptide solution was incubated with anti-Khib antibody-conjugated agarose beads (PTM-804, PTM Bio, Hangzhou, China) at 4 °C overnight. Negative enrichment controls were performed by omitting the anti-Khib antibody. The beads were then washed four times with NETN buffer and twice with ultrapure water. Bound peptides were eluted with 0.1% trifluoroacetic acid, vacuum-dried, and desalted using C18 ZipTips(Merck, Darmstadt, Germany).

### 2.3. LC-MS/MS Analysis and Database Search 

The peptides were dissolved in 0.1% formic acid and separated on a home-made reversed-phase column (15 cm × 100 μm i.d.) using a Vanquish Neo UPLC system (Thermo Fisher Scientific). The gradient of solvent B (0.1% formic acid, 80% acetonitrile) at 400 nL/min was: 0–0.5 min, 4% B; 0.5–0.6 min, 4–8% B; 0.6–13.6 min, 8–22.5% B; 13.6–20.5 min, 22.5–35% B; 20.5–20.9 min, 35–55% B; 20.9–21.4 min, 55–99% B; 21.4–22.6 min, 99% B. Analysis was performed on an Orbitrap Astral mass spectrometer (Thermo Fisher Scientific) with a nano-electrospray ion source at 1900 V. Full MS scans (380–980 *m*/*z*) were acquired at 240,000 resolution. MS/MS scans were acquired at 80,000 resolution with HCD fragmentation at 25% NCE. DIA data were processed using Spectronaut (version 18, Biognosys, Schlieren, Switzerland) against the *Vibrio alginolyticus*_40B_674977_PR_20220406.fasta database which corresponds to strain 40B (NCBI RefSeq: GCF_000176055.1; GenBank: GCA_000176055.1) and contains 4340 protein entries. Trypsin/P was set as the enzyme with up to 4 missed cleavages. Carbamidomethyl on Cys was fixed; acetylation on protein N-terminus, oxidation on Met, and Khib on Lys were variable modifications. FDR was set to <1% at the protein, peptide, and PSM levels. Site localization probability was set to >0.75, and only modified sites identified with at least one unique peptide were retained. The use of the 40B database instead of the HY9901 genome is a limitation, as strain-specific peptides may have been missed. The analysis was performed using the standard pipeline of the service provider (PTM Bio), which was established before the HY9901 genome became publicly available.

### 2.4. Bioinformatics Analysis

Functional annotation and enrichment analyses of Khib-modified proteins were performed using Gene Ontology (GO), Clusters of Orthologous Groups (COG), and Kyoto Encyclopedia of Genes and Genomes (KEGG) databases. GO and KEGG enrichment analyses were conducted using eggNOG-mapper (version 2.1.6) and KOBAS (version 3.0). GO terms and KEGG pathways with a *p*-value < 0.05 were considered significantly enriched, and Benjamini–Hochberg correction was applied for multiple testing. Subcellular localization analysis was performed using PSORTb (version 3.0) to predict the cellular distribution of Khib-modified proteins.

Motif analysis of Khib-modified peptides was performed using the MoMo tool (http://meme-suite.org/tools/momo, accessed on 1 October 2026) based on the motif-x algorithm. Peptide sequences containing 10 amino acids upstream and downstream of each modified lysine residue were extracted and analyzed. The background set consisted of all tryptic peptides from the *V. alginolyticus* proteome. Protein–protein interaction networks of Khib-modified proteins were constructed using STRING (version 11.5).

### 2.5. Gene Cloning and Mutant Strain Construction

The *cobQ* gene was amplified from the genomic DNA of *V. alginolyticus* HY9901 using specific primers *cobQ*-F1/R1 (see Table 3). The PCR product was ligated into the pMD18-T vector and verified by sequencing (Sangon Biotech, Shanghai, China). To construct the *cobQ* deletion mutant, the upstream and downstream homologous arms of *cobQ* were amplified and cloned into the suicide plasmid pLP12. The deletion fragment was approximately 780 bp, corresponding to the full-length coding sequence of *cobQ*. The recombinant plasmid was introduced into Escherichia coli and subsequently transferred into *V. alginolyticus* HY9901 by conjugation. Single-crossover mutants were selected on TSA plates containing chloramphenicol. Double-crossover mutants were counter-selected on TSA plates containing 10% sucrose. The *cobQ* deletion mutant was verified by PCR using specific primers Δ*cobQ*-F/R (see Table 3) and confirmed by Sanger sequencing. To construct the complemented strain C-*cobQ*, the *cobQ* gene together with its native promoter was cloned into the pBBR1-MCS plasmid (a broad-host-range vector) via the BamHI and HindIII (Takara, Beijing, China) restriction sites. The construct was introduced into the Δ*cobQ* mutant by conjugation. Positive colonies were verified by PCR using specific primers C-*cobQ*-F/R (see Table 3) and sequencing. The expression of *cobQ* in the complemented strain was expected to be driven by its native promoter, but the actual expression level was not verified by RT-qPCR or Western blot.

### 2.6. CobQ de-Khib Activity Assay

For in vivo de-Khib assay, global Khib modification levels in the wild-type strain HY9901 and the Δ*cobQ* deletion mutant were analyzed by Western blot. Total protein (20 μg per lane) was separated by SDS-PAGE and transferred onto a PVDF membrane (Yeasen, Shanghai, China). The membrane was incubated with anti-Khib antibody (1:1000, PTM Bio, PTM0804) at 4 °C overnight. RNA polymerase β subunit (RNAP β, BioLegend, San Diego, CA, USA) served as the loading control. HRP-conjugated secondary antibody was used for detection by ECL chemiluminescence.

For in vitro de-Khib assay, recombinant His-tagged CobQ protein was expressed in *E. coli* BL21(DE3) and purified using Ni-NTA affinity chromatography. The Khib-modified BSA substrate was prepared by mixing 1 mL of BSA (1 mg/mL) with 400 μL of 2-hydroxyisobutyryl-CoA (1 mg/mL, Yuanye, Shanghai, China), corresponding to a molar ratio of approximately 1:31 (BSA: Khib-CoA), and incubating at 37 °C for 3 h. After incubation, the sample was desalted. The extent of modification and the number of modification sites were not further characterized, which is acknowledged as a limitation. Purified CobQ protein (0.2 μg) was incubated with 10 μg of Khib-modified BSA in reaction buffer (150 mM Tris-HCl, pH 8.0, 10% glycerol) at 37 °C for 4 h. To determine whether CobQ-mediated de-Khib activity requires common deacylase cofactors, reactions were supplemented with Zn^2+^, ATP, or NAD^+^/NAM, representing the major classes of known deacylases. The reaction groups were set up as follows: (1) Khib-modified BSA alone (no CobQ); (2) Khib-modified BSA + CobQ; (3) Khib-modified BSA + CobQ + Zn^2+^ (1 mM); (4) Khib-modified BSA + CobQ + ATP (1 mM); (5) Khib-modified BSA + CobQ + NAD^+^ (1 mM) + NAM (10 mM). After incubation, the Khib level of the BSA was detected by Western blot using anti-Khib antibody. Positive controls (a known deacylase), heat-inactivated CobQ, and unrelated protein controls were not included, which is acknowledged as a limitation.

### 2.7. Genetic Stability Test

*V. alginolyticus* strains HY9901, Δ*cobQ,* and C-*cobQ* were serially passaged for 30 generations in TSB medium at 28 °C with shaking at 200 rpm (12 h per generation). Genomic DNA was extracted from the 30th generation. The stability of the Δ*cobQ* mutation was verified by PCR using primers Δ*cobQ*-yz-F/R (see Table 3). The retention of the complementary plasmid in C-*cobQ* was verified by PCR using primers pBBR1-YZ-F/R (see Table 3). In addition, the *cobQ* gene in the plasmid was confirmed by sequencing using primers C-*cobQ*-F/R (see Table 3).

### 2.8. Growth Curve Experiment

Strains HY9901, Δ*cobQ*, and C-*cobQ* were cultured overnight in TSB medium at 28 °C with shaking at 200 rpm. The OD_600_ was adjusted to 0.5, and cultures were transferred at a 1:100 ratio into fresh TSB medium. OD_600_ values were measured at 2 h intervals for 24 h. Growth curves were plotted as OD_600_ versus time. For this assay, three independent overnight cultures were used as biological replicates (*n* = 3), and each culture was measured individually. Data were analyzed by two-way ANOVA.

### 2.9. Biofilm Formation Assay

Strains HY9901, Δ*cobQ*, and C-*cobQ* were cultured in TSB to an OD_600_ of 0.5. Bacterial suspension (200 μL/well) was inoculated into 96-well plates with three technical replicates per strain, and TSB medium alone served as the negative control. Plates were incubated statically at 28 °C. At 6, 12, 24, 48, and 72 h, wells were washed three times with PBS, fixed with 200 μL of methanol for 20 min, and air-dried. The wells were then stained with 200 μL of 0.1% crystal violet solution for 15 min, rinsed gently with tap water, and air-dried. Bound crystal violet was dissolved in 200 μL of 95% ethanol for 30 min, and the OD_570_ was measured. The results are presented as descriptive observations, as independent biological replicates were not performed.

### 2.10. Extracellular Enzyme Assay

HY9901, Δ*cobQ*, and C-*cobQ* (1 mL, OD_600_ = 0.5) were spread onto TSA plates covered with sterile cellophane and incubated at 28 °C for 18 h (the optimal time point for extracellular protease production based on preliminary experiments). Bacteria were rinsed off with PBS and centrifuged at 12,000× *g* for 10 min at 4 °C. The supernatant was filtered through a 0.22 μm membrane. Protease activity was measured using the azocasein method. Absorbance was read at 422 nm. All experiments were performed with three independent biological replicates (*n* = 3). Data are presented as mean ± standard deviation (SD).

### 2.11. Swimming Motility Assay

TSA plates were prepared with agar concentrations of 0.3%, 0.4%, and 0.5%. Strains HY9901, Δ*cobQ*, and C-*cobQ* were streaked on TCBS plates for isolation. A single colony was picked with a sterile toothpick and inoculated vertically into the center of each TSA plate. Plates were incubated at 28 °C for 18 h, and colony diameters were measured in mm. All experiments were performed with three independent biological replicates (*n* = 3).

### 2.12. Antimicrobial Susceptibility Test

Antimicrobial susceptibility was tested by the disk diffusion method (Kirby-Bauer method). Briefly, strains HY9901 and Δ*cobQ* were cultured to an OD_600_ of 0.5 and evenly spread onto Mueller-Hinton agar (MHA) plates. Antibiotic disks were placed onto the plates and incubated at 28 °C for 18–24 h. Inhibition zone diameters were measured in mm and are presented as descriptive observations. S/I/R classifications were not assessed due to the lack of validated species-specific breakpoints for all tested antibiotics. All experiments were performed with three technical replicates (*n* = 3). The complemented strain C-*cobQ* was not included in this assay due to limited sample availability.

## 3. Result and Discussion

### 3.1. Global Identification of Khib Sites in V. alginolyticus

To systematically characterize lysine 2-hydroxyisobutyrylation in *V. alginolyticus*, Khib-modified peptides were enriched using anti-Khib antibody-conjugated beads and analyzed by LC-MS/MS. A total of 15,195 Khib modification sites were identified in 2848 proteins, indicating that Khib is widely distributed in the proteome of *V. alginolyticus*.

**Figure 1:**
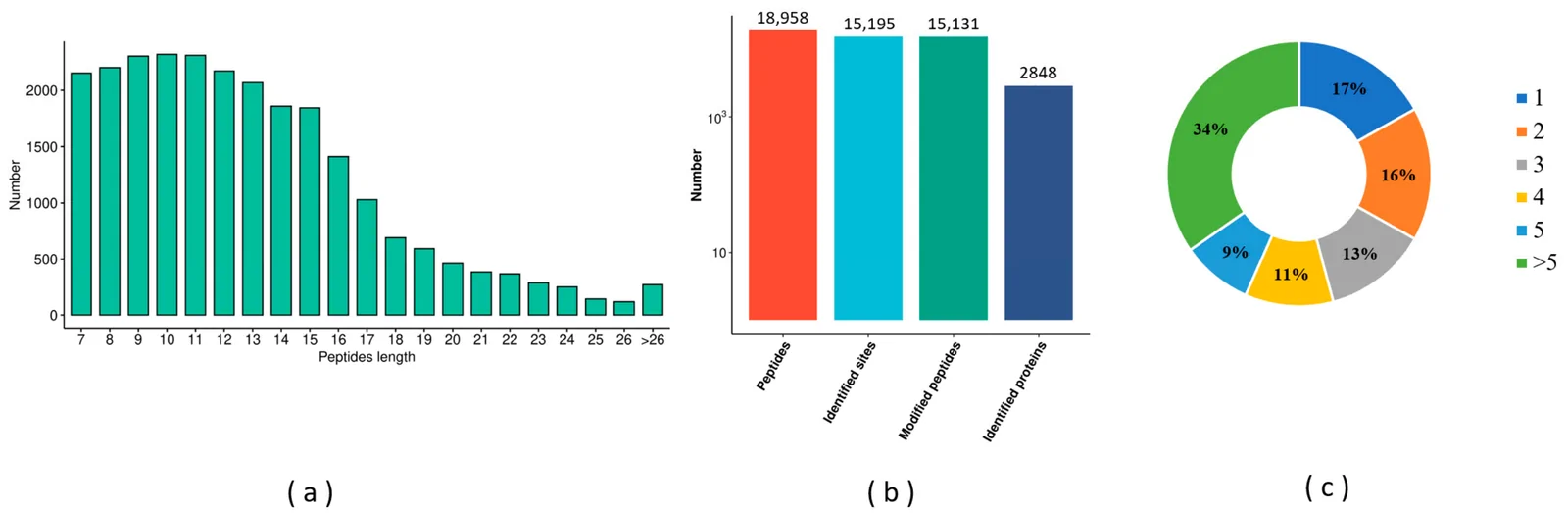
Profile of *Vibrio alginolyticus* Khib proteome. (**a**) Lysine hydroxyisobutyrylated peptide distribution based on length. (**b**) Summary of identified total peptides (18,958), Khib-modified peptides (15,131), Khib-modified sites (15,195), and Khib-modified proteins (2848). (**c**) Pie chart showing the distribution of Khib sites per protein: 1 site (17%), 2 sites (16%), 3 sites (13%), 4 sites (11%), 5 sites (9%), and >5 sites (34%).

The length distribution of identified Khib-modified peptides was mainly concentrated between 7 and 20 amino acids (Figure 1a), which is consistent with the general characteristics of tryptic peptides suitable for mass spectrometric identification [15]. A total of 18,958 peptides were identified, among which 15,131 were Khib-modified peptides, corresponding to 15,195 non-redundant modification sites distributed across 2848 proteins (Figure 1b). As shown in the pie chart (Figure 1c), 17% of the modified proteins carried only one Khib site, 16% carried two sites, 13% carried three sites, 11% carried four sites, 9% carried five sites, and 34% contained more than five sites. These results indicate that Khib is widely distributed in the proteome of *V. alginolyticus*, with the majority of modified proteins carrying a limited number of Khib sites, and a smaller subset containing more than five sites.

### 3.2. Functional Annotation of Khib-Modified Proteins

To explore the potential biological functions of Khib-modified proteins in *V. alginolyticus*, GO, COG, KEGG, and subcellular localization analyses were performed.

**Figure 2:**
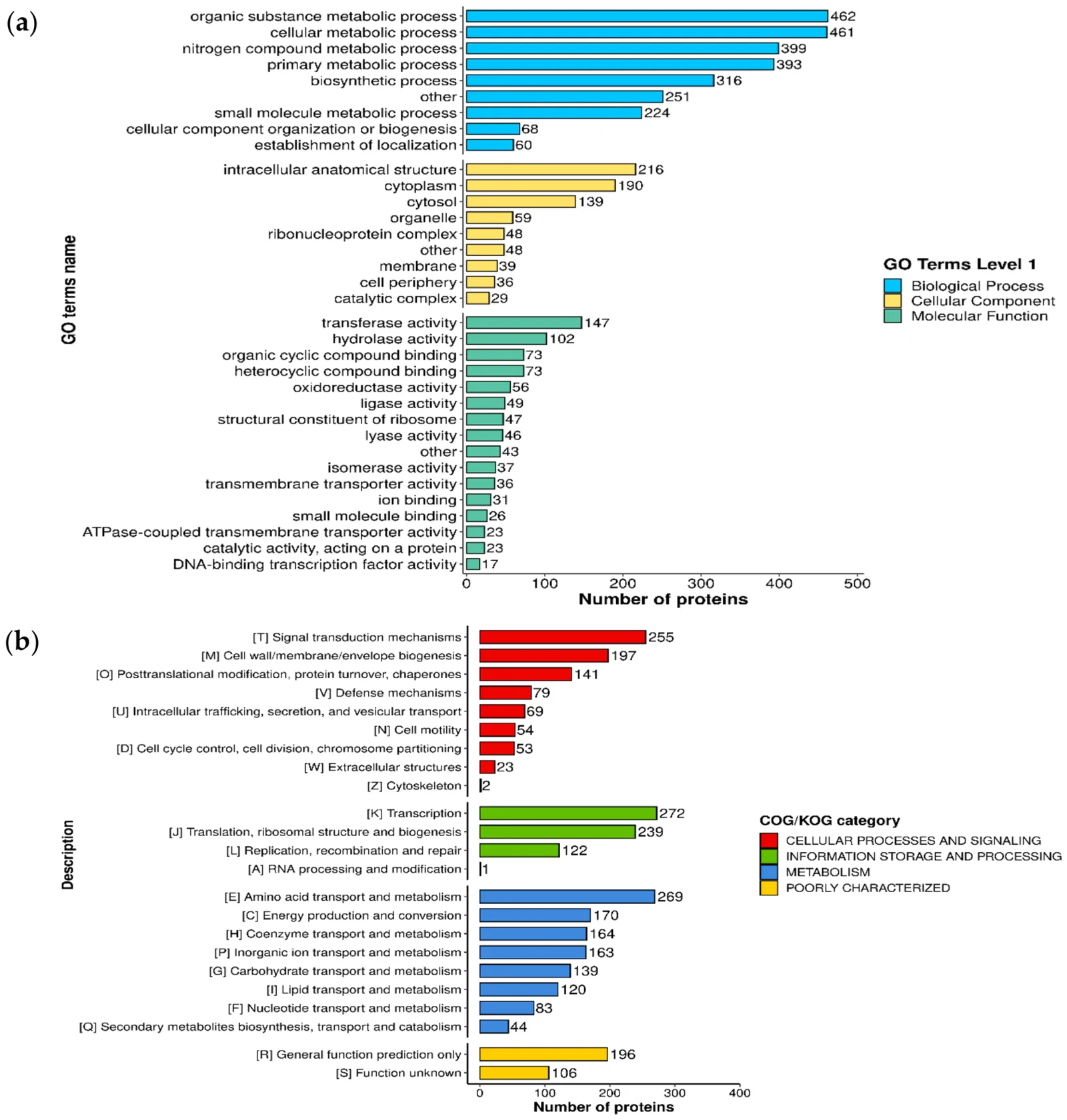

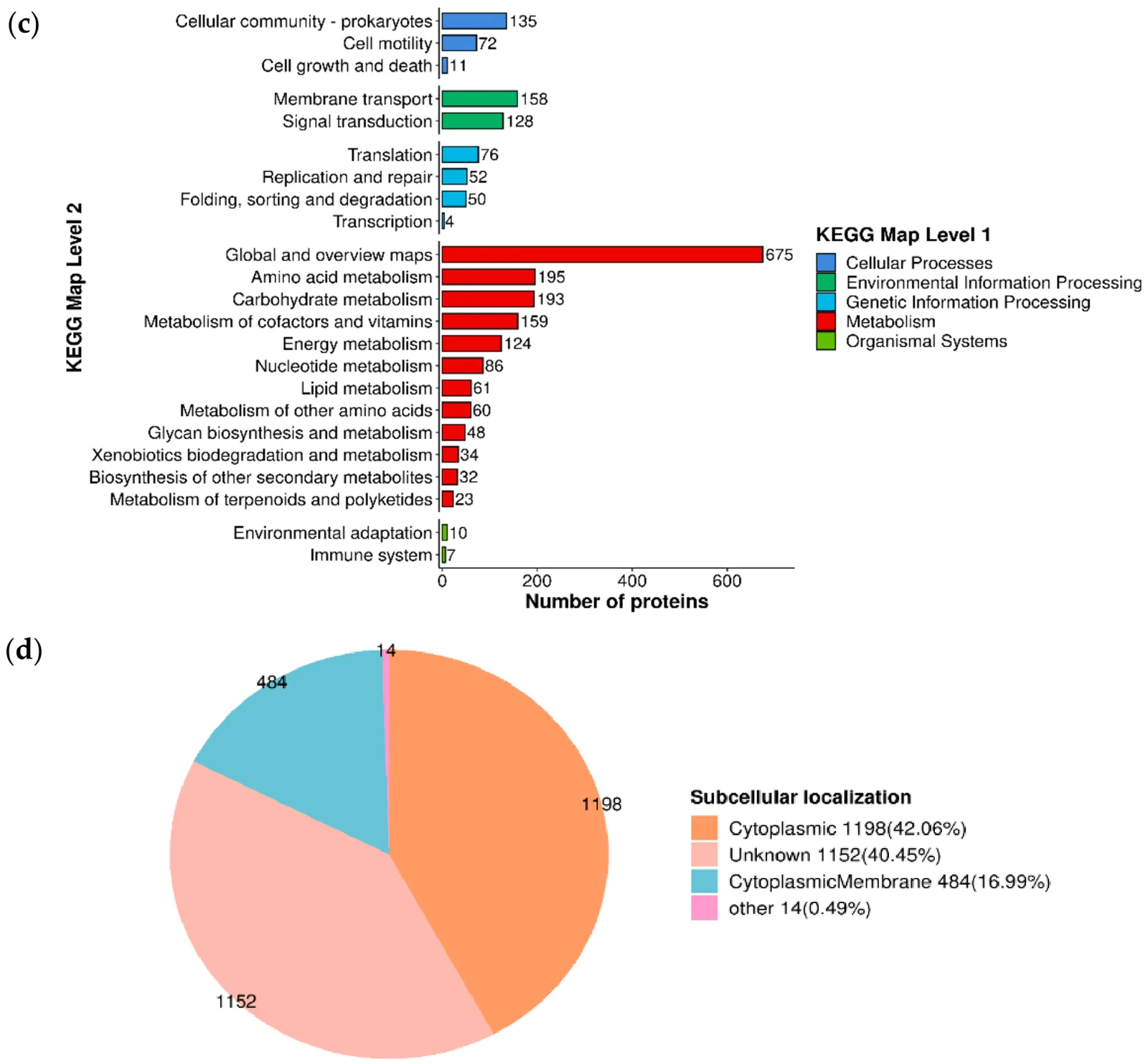
Functional annotation and enrichment of hydroxyisobutyrylated proteins in *V. alginolyticus*: (**a**) GO analysis; (**b**) COG analysis; (**c**) KEGG pathway analysis; (**d**) subcellular localization analysis. The letters at the beginning of each term in (**b**) indicate the functional category codes. Note: Percentages may not sum to 100% due to rounding.

GO analysis showed that Khib-modified proteins were mainly enriched in metabolic processes in the biological process category. These processes included organic substance metabolic process, cellular metabolic process, nitrogen compound metabolic process, and primary metabolic process. In the cellular component category, Khib-modified proteins were mainly associated with intracellular anatomical structures, cytoplasm, and cytosol. In the molecular function category, transferase activity, hydrolase activity, organic cyclic compound binding, and heterocyclic compound binding were prominently represented (Figure 2a). These results suggest that Khib-modified proteins are enriched in metabolic pathways and enzymatic functions in *V. alginolyticus*.

COG classification further showed that Khib-modified proteins were distributed across multiple functional categories, including amino acid transport and metabolism, translation, ribosomal structure and biogenesis, transcription, signal transduction mechanisms, cell wall/membrane/envelope biogenesis, and intracellular trafficking and secretion (Figure 2b). Among these categories, amino acid metabolism and carbohydrate metabolism contained relatively large numbers of Khib-modified proteins, suggesting that Khib-modified proteins are enriched in amino acid and carbohydrate metabolism pathways.

KEGG pathway analysis showed that many Khib-modified proteins were assigned to global and overview metabolic maps, amino acid metabolism, carbohydrate metabolism, energy metabolism, membrane transport and signal transduction (Figure 2c). These findings indicate that Khib-modified proteins are frequently found in pathways related to core physiological processes of *V. alginolyticus*. Notably, several Khib-modified proteins were associated with signal transduction and membrane biosynthesis, suggesting a potential link between Khib modification and virulence-associated biological processes based on annotation.

Subcellular localization analysis showed that Khib-modified proteins were mainly distributed in the cytoplasm and cytoplasmic membrane. A considerable proportion of proteins were assigned to unknown localization categories (Figure 2d), indicating that many Khib-modified proteins remain functionally uncharacterized. These proteins may represent important candidates for future studies on Khib-mediated regulation in *V. alginolyticus*.

### 3.3. Motif Analysis of Khib Sites

**Figure 3:**
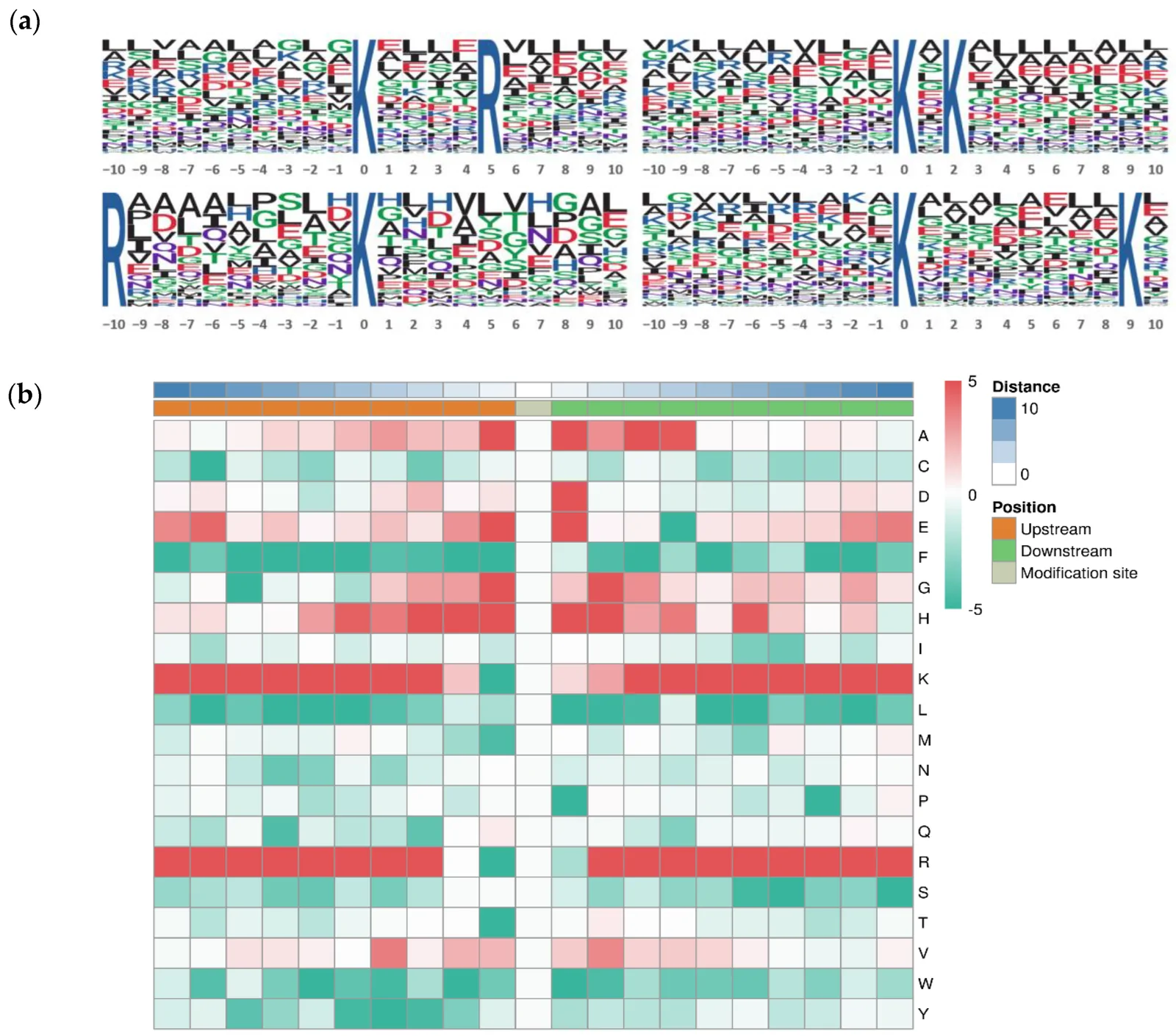
Motif analysis of Khib sites. (**a**) Sequence logos of motifs (*p* < 0.000001) were identified using MoMo software (version 5.5.7). (**b**) Heatmap illustrating the distribution frequency of various types of amino acid residues surrounding Khib sites. The single-letter amino acid codes (e.g., A, C, D, E, F, G, H, I, K, L, M, N, P, Q, R, S, T, V, W, Y) represent the 20 standard amino acids.

Motif analysis of Khib-modified peptides was performed using the MoMo tool based on the motif-x algorithm. Peptide sequences consisting of 10 amino acids upstream and downstream of all identified sites were analyzed, with all potential modification site sequences in the species used as the background. A total of 37 sequence motifs were matched (Figure 3a). A heatmap was generated to visualize the degree of change score (DS) for amino acid frequency near modification sites (Figure 3b), calculated as DS = −Log^10^(*p*.value) × sign(diff.percent).

### 3.4. Protein–Protein Interaction Network of Khib-Modified Proteins

**Figure 4:**
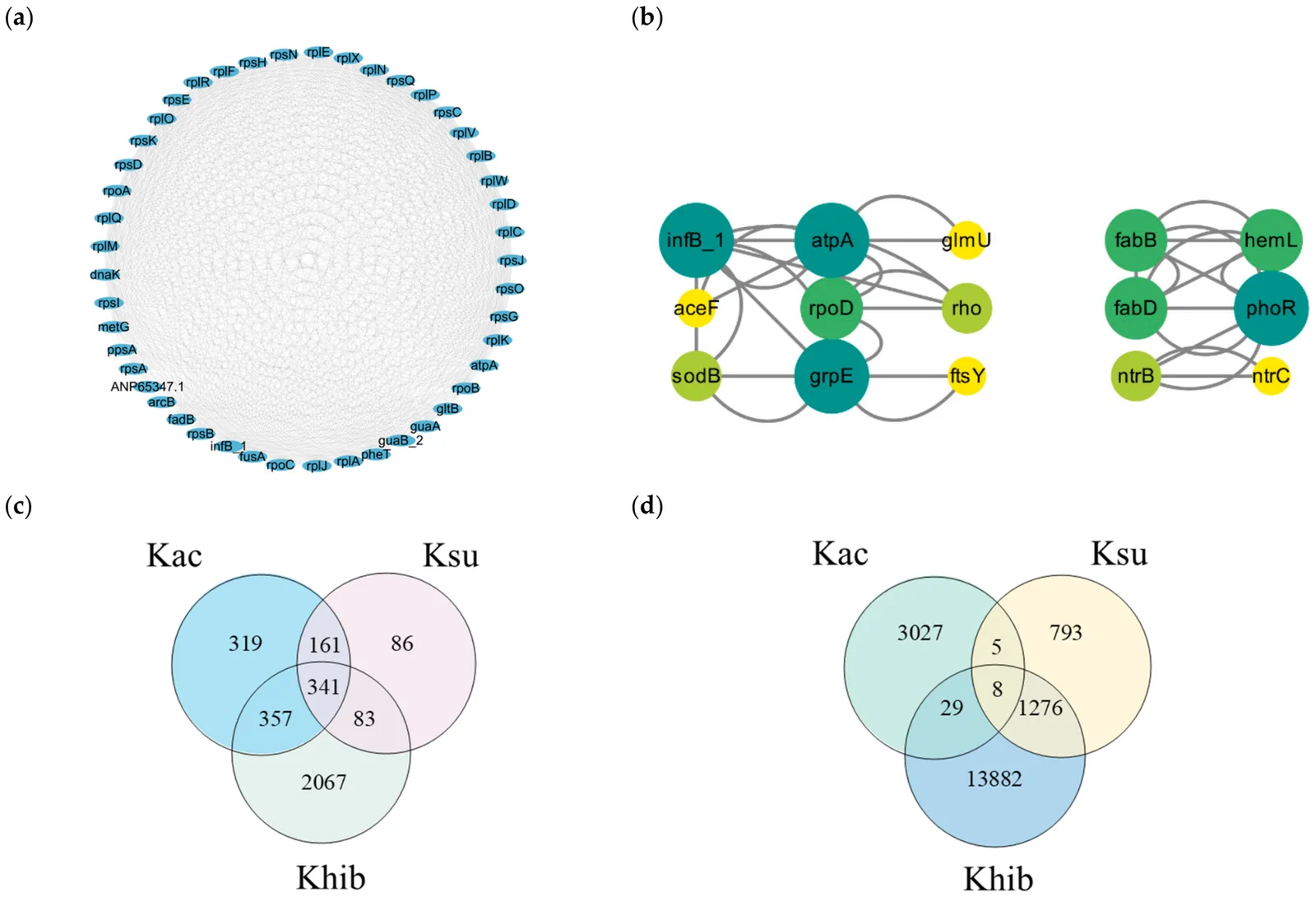
Protein–protein interaction network of lysine Khib-modified proteins and comparison of different lysine acylation modifications. (**a**) Khib-modified proteins PPI network; (**b**) protein–protein interaction network of lysine Khib-modified virulence proteins; (**c**) comparison of the numbers of Khib-modified proteins with previously reported lysine acetylation (Kac) and lysine succinylation (Ksu) datasets in *V. alginolyticus*. (**d**) Comparison of the numbers of Khib-modified sites with Kac and Ksu datasets in *V. alginolyticus*.

To explore the potential functional connections among Khib-modified proteins, a protein–protein interaction network was constructed using STRING. The Khib-modified proteins formed a complex interaction network (Figure 4a), suggesting that Khib-modified proteins may be functionally connected across multiple biological processes.

Further analysis of annotated virulence-associated proteins showed that several Khib-modified proteins potentially linked to virulence-related processes, including InfB (translation initiation factor), AtpA (ATP synthase), GlmU (cell wall synthesis), FabB/FabD (fatty acid biosynthesis), HemL (heme biosynthesis), AceF (metabolism), RpoD/Rho (transcription regulation), PhoR (signal transduction), SodB (oxidative stress response), GrpE (protein folding), FtsY (protein secretion), and NrrB/NrrC (two-component regulation) (Figure 4b). These results suggest that Khib-modified proteins may be functionally associated with these processes. However, as the Protein–Protein interaction (PPI) analysis is based on database prediction and known interaction information, the biological relevance of these interactions should be verified experimentally in future studies.

In addition, Khib modification was compared with previously reported lysine acetylation and lysine succinylation datasets in *V. alginolyticus*. The comparison showed that Khib modification displayed distinct characteristics in terms of the number of modified proteins (Figure 4c) and modification sites (Figure 4d). These findings suggest that different lysine acylation modifications may form a complex PTM regulatory network in *V. alginolyticus*.

### 3.5. Protein-Level Detectio of Selected Khib-Modified Proteins

**Figure 5:**
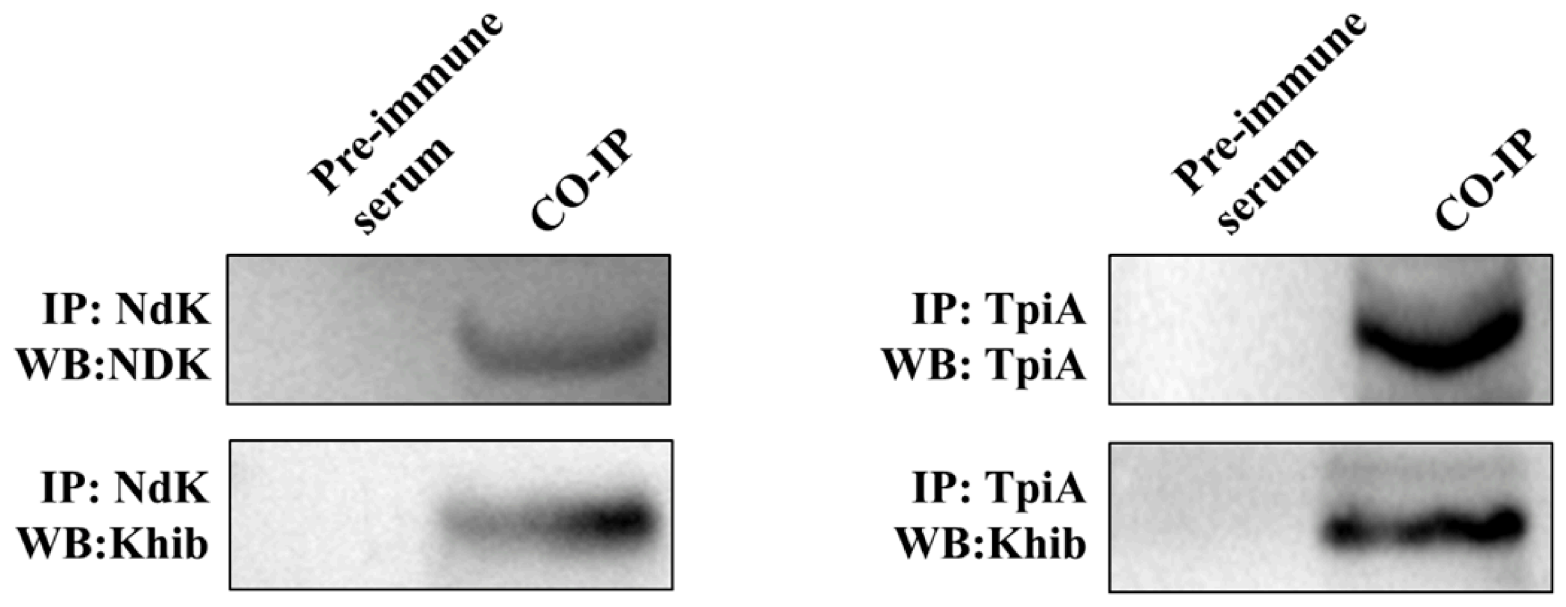
Protein-level detection of Khib-modified TpiA and Ndk in *V. alginolyticus* by co-immunoprecipitation (Co-IP) and Western blot (WB). Immunoprecipitation was performed using anti-TpiA or anti-Ndk antibodies, followed by immunoblotting with anti-TpiA/Ndk or anti-Khib antibodies. The pre-immune serum served as a negative control. The top panels show the expression of TpiA and Ndk, and the bottom panels confirm their Khib modification. The data provide protein-level evidence for Khib on TpiA and Ndk, consistent with the proteomic identification, but do not constitute a strict orthogonal validation of the entire Khib dataset. Input controls were not included in this experiment; this limitation is acknowledged in the Discussion.

TpiA (triosephosphate isomerase) and Ndk (nucleoside diphosphate kinase) were selected for protein-level analysis because they represent high-confidence Khib targets involved in central metabolism. Immunoprecipitation combined with Western blot was performed to verify their Khib modification (Figure 5). Immunoprecipitation was performed using anti-TpiA or anti-Ndk antibodies, followed by immunoblotting with anti-Khib antibodies. Pre-immune serum was used as a negative control. The results showed that both TpiA and Ndk could be detected in the Khib-enriched protein fraction, supporting the presence of Khib modification on these proteins (Figure 5). However, Input controls were not included, which limits the interpretation of this Co-IP experiment. These results are consistent with the proteomic identification and provide protein-level evidence for Khib on TpiA and Ndk. However, it does not confirm the specific modification sites identified by LC-MS/MS. Site-level validation would require targeted MS/MS (e.g., PRM) or site-specific mutagenesis in future studies.

### 3.6. Construction and Verification of ΔcobQ and C-cobQ Strains

**Figure 6:**
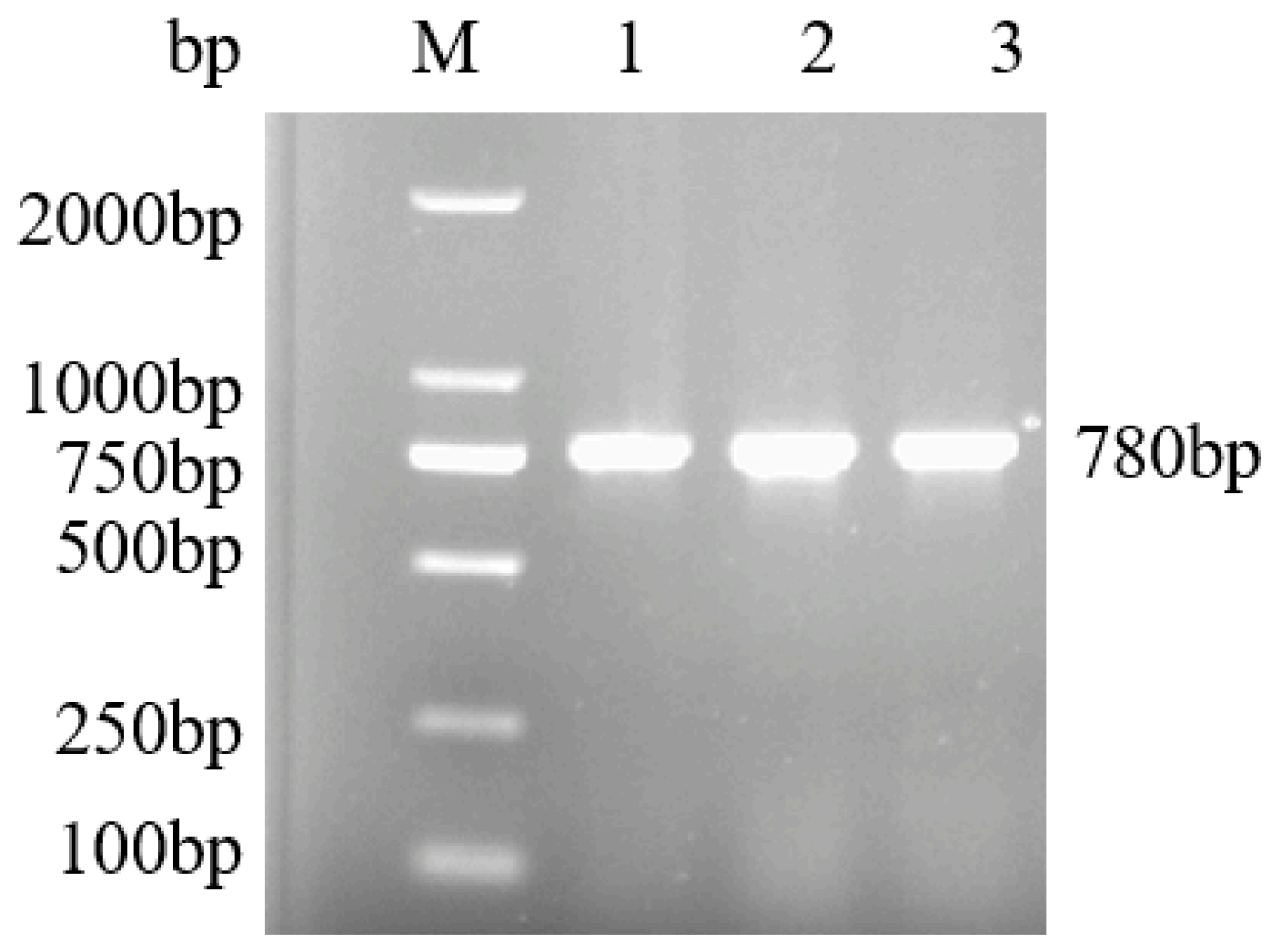
Cloning of the *cobQ* gene. Lanes 1–3: *cobQ*.

**Figure 7:**
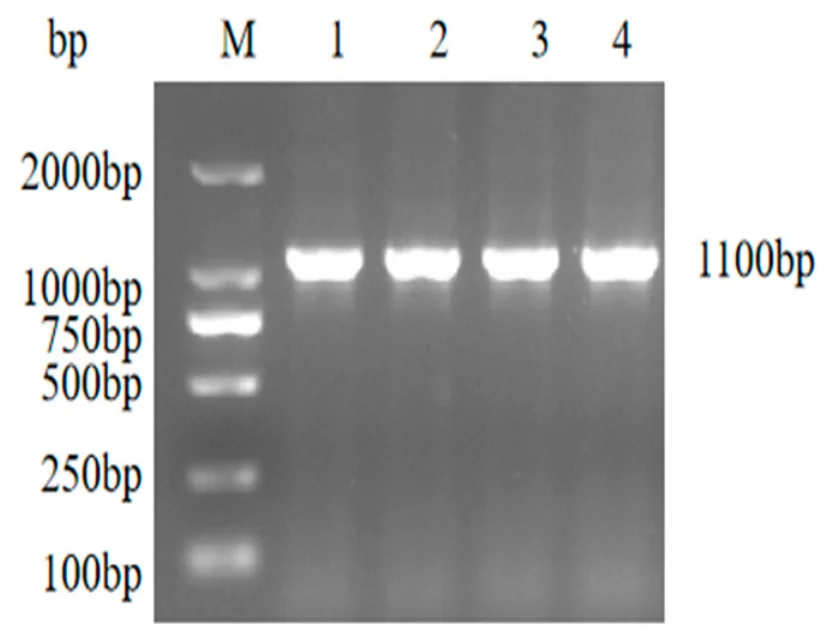
Construction of *cobQ* deletion mutant of *Vibrio alginolyticus.* Lanes 1–4: Δ*cobQ*.

**Figure 8:**
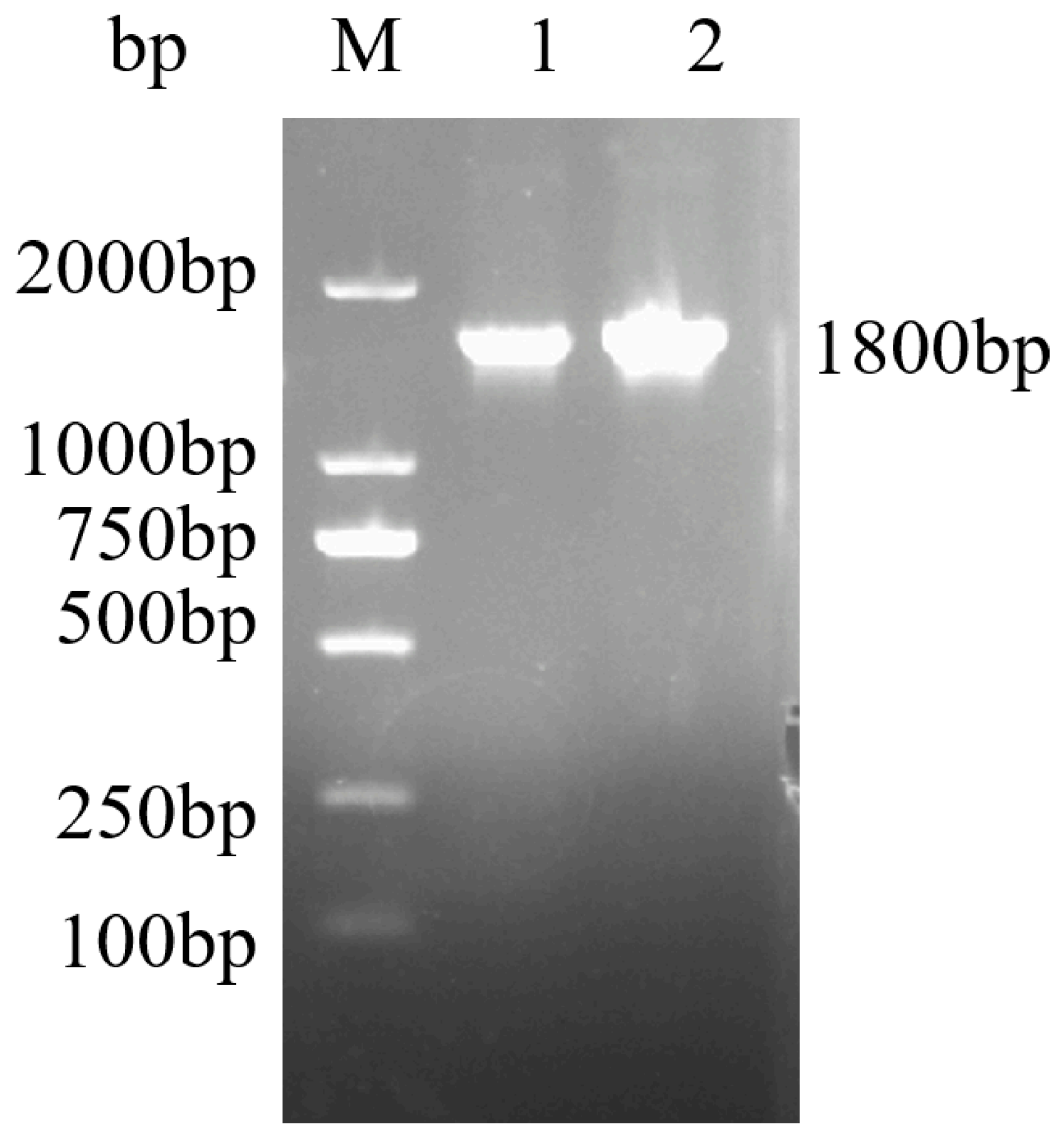
Construction of *cobQ*-complemented strain C-*cobQ* of *Vibrio alginolyticus.* Lanes 1–2: C-*cobQ*.

To construct the *cobQ* deletion mutant, the upstream and downstream homologous arms of *cobQ* were amplified and cloned into the suicide plasmid pLP12. A deletion fragment of approximately 780 bp within the *cobQ* coding sequence was generated. The recombinant plasmid was introduced into *E. coli* S17-1 and transferred into *V. alginolyticus* HY9901 by conjugation. The deletion mutant (Δ*cobQ*) was verified by PCR (see Table 3) and Sanger sequencing. To construct the plasmid-complemented strain C-*cobQ*, the *cobQ* gene (Figure 6, lanes 1–3) together with its native promoter was cloned into the pBBR1-MCS plasmid (a broad-host-range vector) and introduced into the Δ*cobQ* mutant. Positive colonies were verified by PCR (see Table 3) and Sanger sequencing. PCR verification showed specific bands of approximately 1100 bp for Δ*cobQ* (Figure 7, lanes 1–4) and 1800 bp for C-*cobQ* (Figure 8, lanes 1–2), confirming the presence of the expected constructs.

### 3.7. CobQ Affects Global Khib Levels

**Figure 9:**
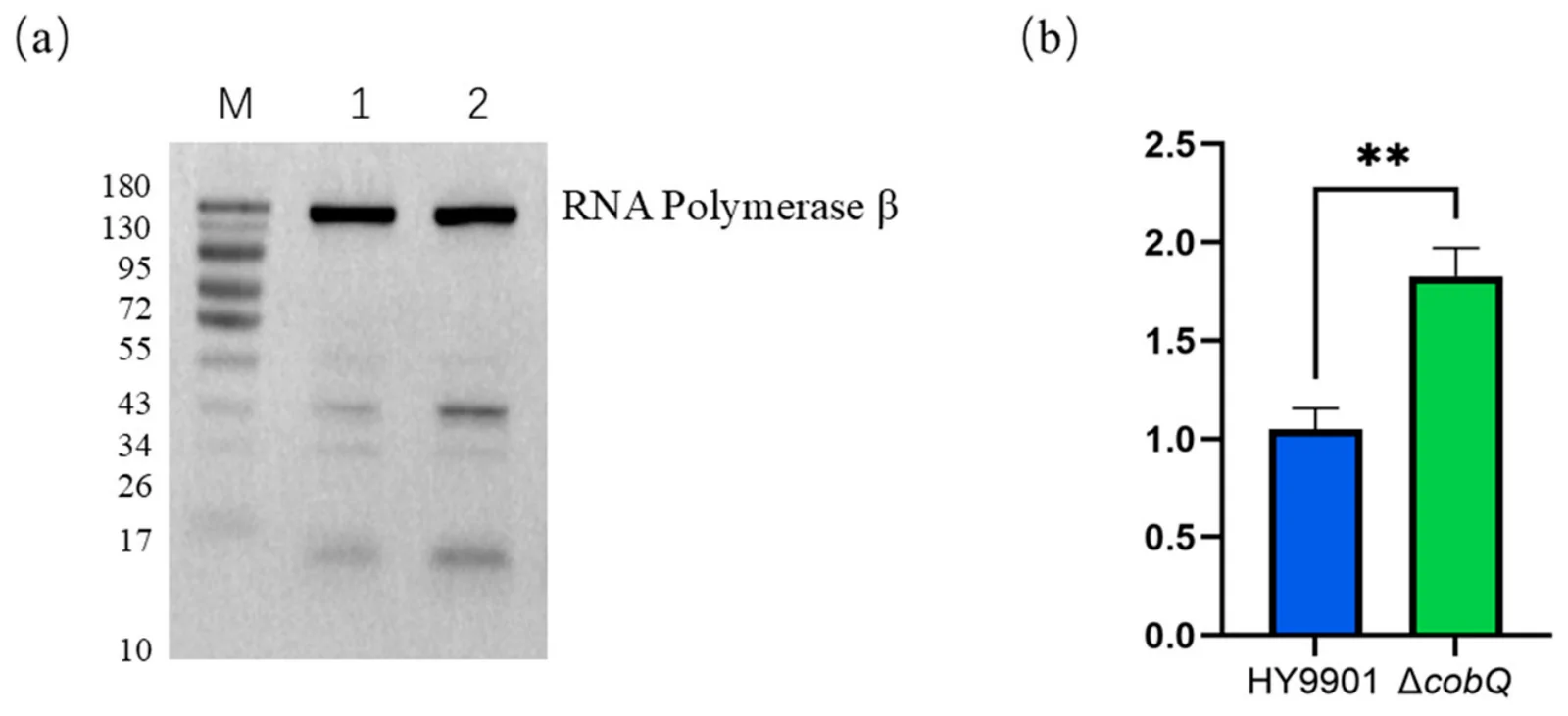
Comparison of global Khib levels between HY9901 (wild-type) and Δ*cobQ* strains. (**a**) Western blot analysis of global Khib modification levels. Lanes 1: HY9901 (wild-type); Lanes 2: Δ*cobQ*. (**b**) Densitometric quantification of Khib signals from three independent experiments (mean ± SD). ** *p* < 0.01 (Student’s *t*-test, Δ*cobQ* vs. wild-type). RNA polymerase β (RNAP β) was used as the loading control.

Western blot analysis revealed that compared to the wild-type strain (HY9901), the Δ*cobQ* strain showed significantly increased global Khib modification levels. RNA polymerase β served as a loading control. However, without the restoration of global Khib levels in the plasmid-complemented strain, this result should be interpreted only as an association between the loss of the *cobQ* locus and a change in the overall Khib signal, rather than as proof of a specific role of CobQ (Figure 9).

**Figure 10:**
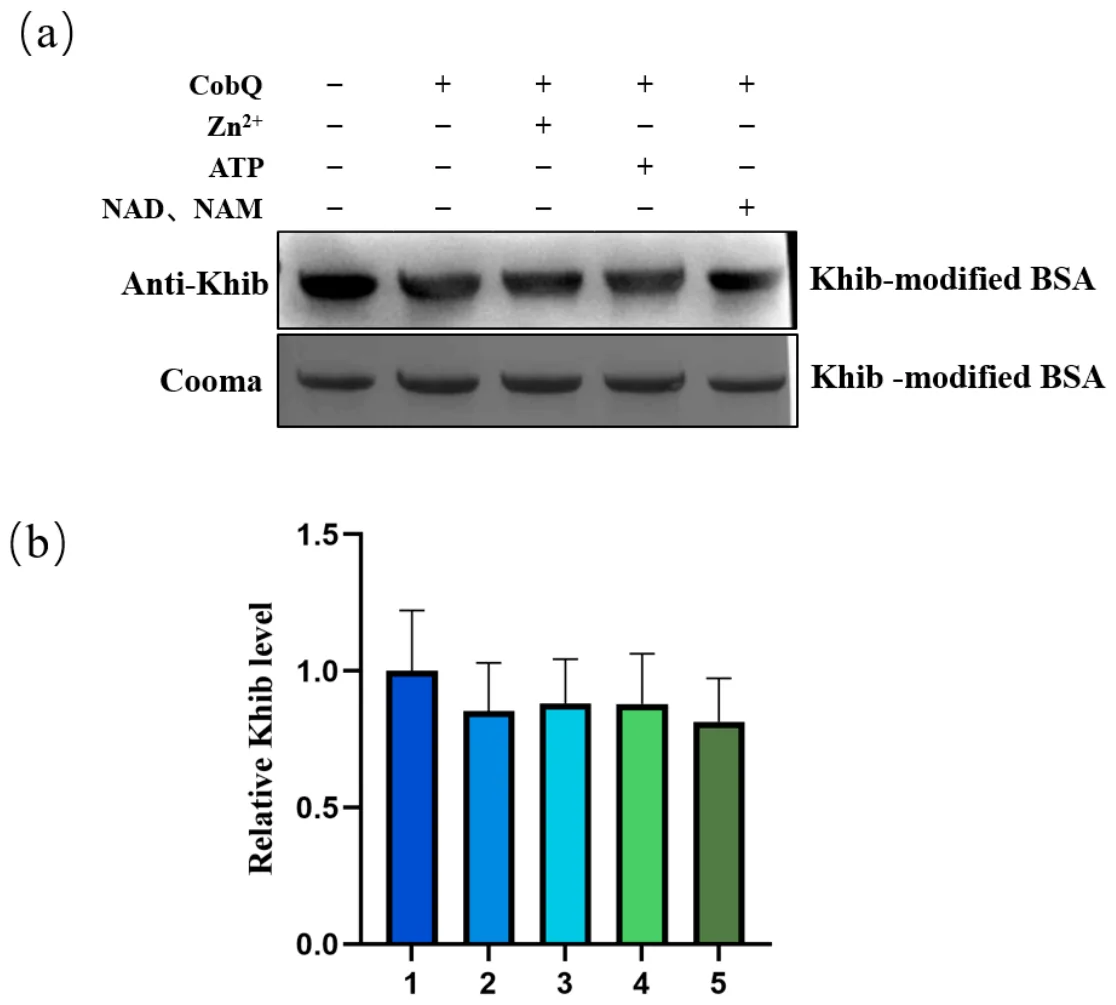
In vitro assay of CobQ with Khib-modified BSA. (**a**) Western blot analysis of Khib levels and corresponding SDS-PAGE with Coomassie blue staining; (**b**) quantification of Khib levels. 1: No CobQ; 2: CobQ; 3: CobQ + Zn^2+^; 4: CobQ + ATP; 5: CobQ + NAD^+^ and NAM.

In vitro de-Khib assays showed no significant difference in Khib levels among the five groups, although a trend toward reduction was observed in the presence of CobQ (Figure 10). As the difference was not statistically significant, these data do not support a direct de-Khib activity of CobQ under the tested conditions. Therefore, these data do not support a conclusion regarding cofactor dependence.

### 3.8. Genetic Stability of the ΔcobQ and C-cobQ Mutants

**Figure 11:**
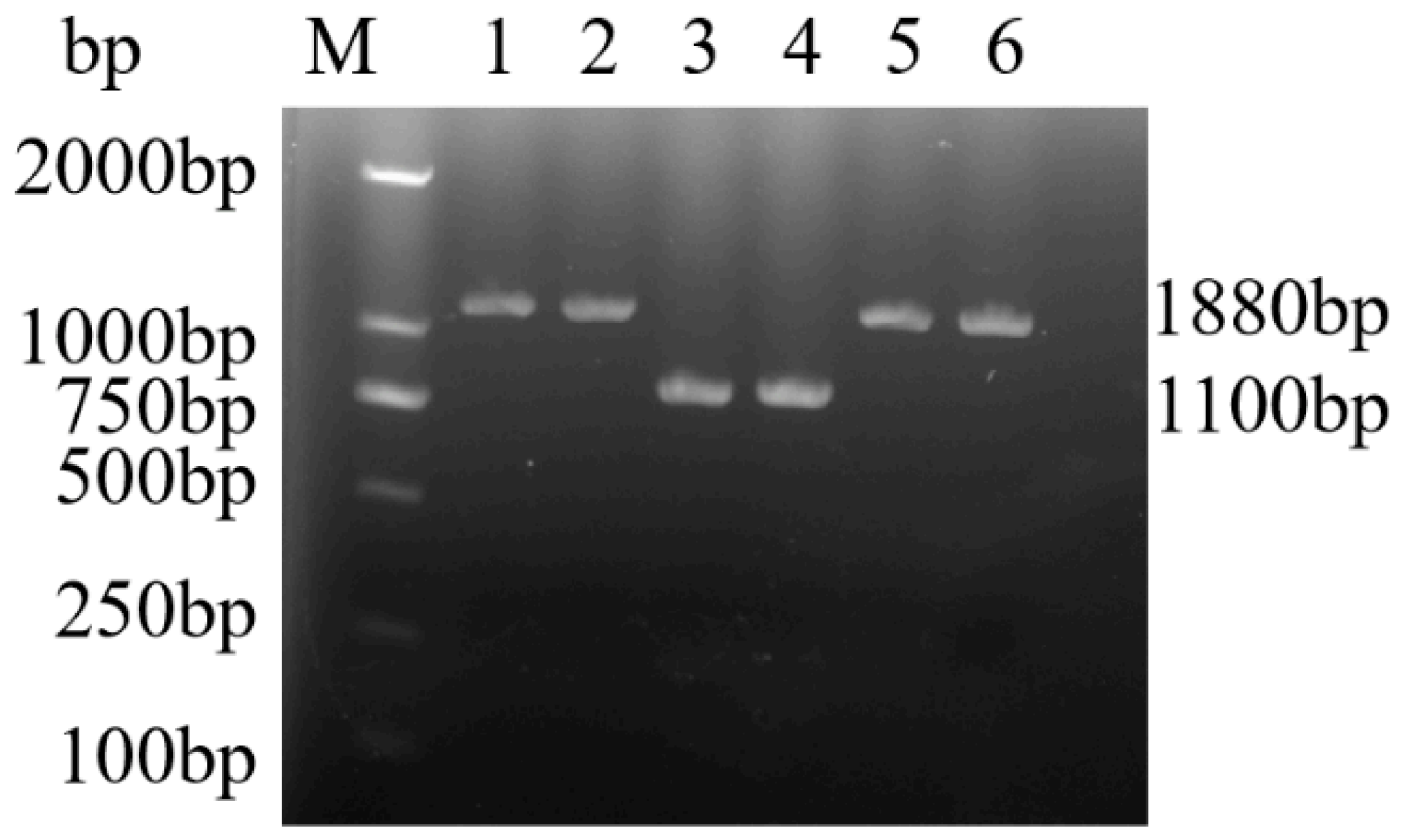
Genetic stability assay of Δ*cobQ* and C-*cobQ*. Lanes 1–2: wild-type strain; Lanes 3–4: Δ*cobQ*; Lanes 5–6: C-*cobQ*.

After 30 serial passages, the wild-type strain produced a characteristic 1880 bp fragment, while the Δ*cobQ* and C-*cobQ* strains stably produced specific bands of 1100 bp, indicating that both mutant strains were genetically stable (Figure 11).

### 3.9. Effect of cobQ Deletion on Bacterial Growth

**Figure 12:**
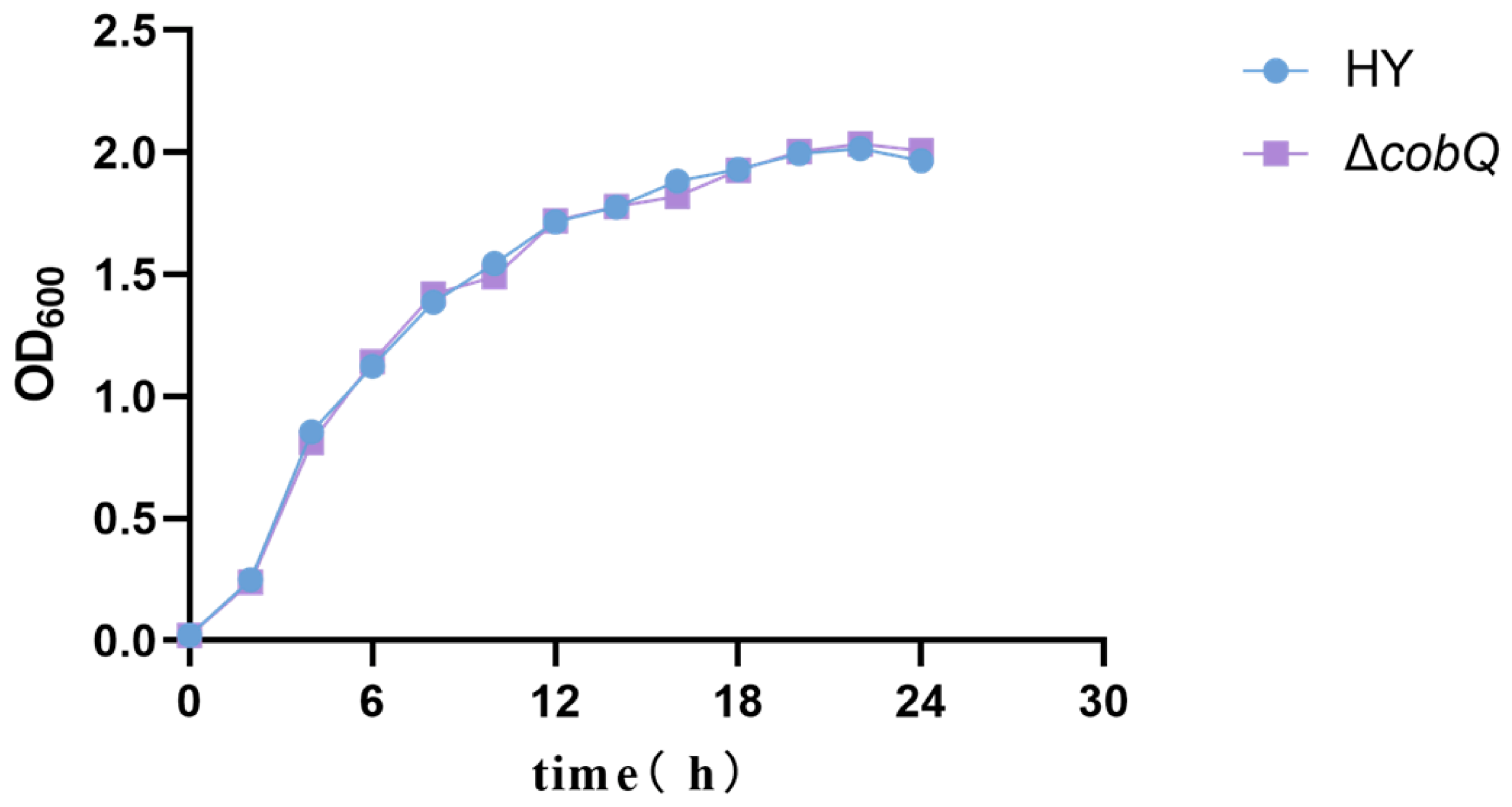
Growth curves of HY9901 and Δ*cobQ* strains.

Growth curve analysis showed no significant difference in growth rates between HY9901 and Δ*cobQ* (*p* > 0.05), indicating that *cobQ* deletion does not affect the growth of *V. alginolyticus* (Figure 12). The plasmid-complemented strain (C-*cobQ*) results are shown in Supplementary Figure S1.

### 3.10. Effect of cobQ Deletion on Biofilm Formation

**Figure 13:**
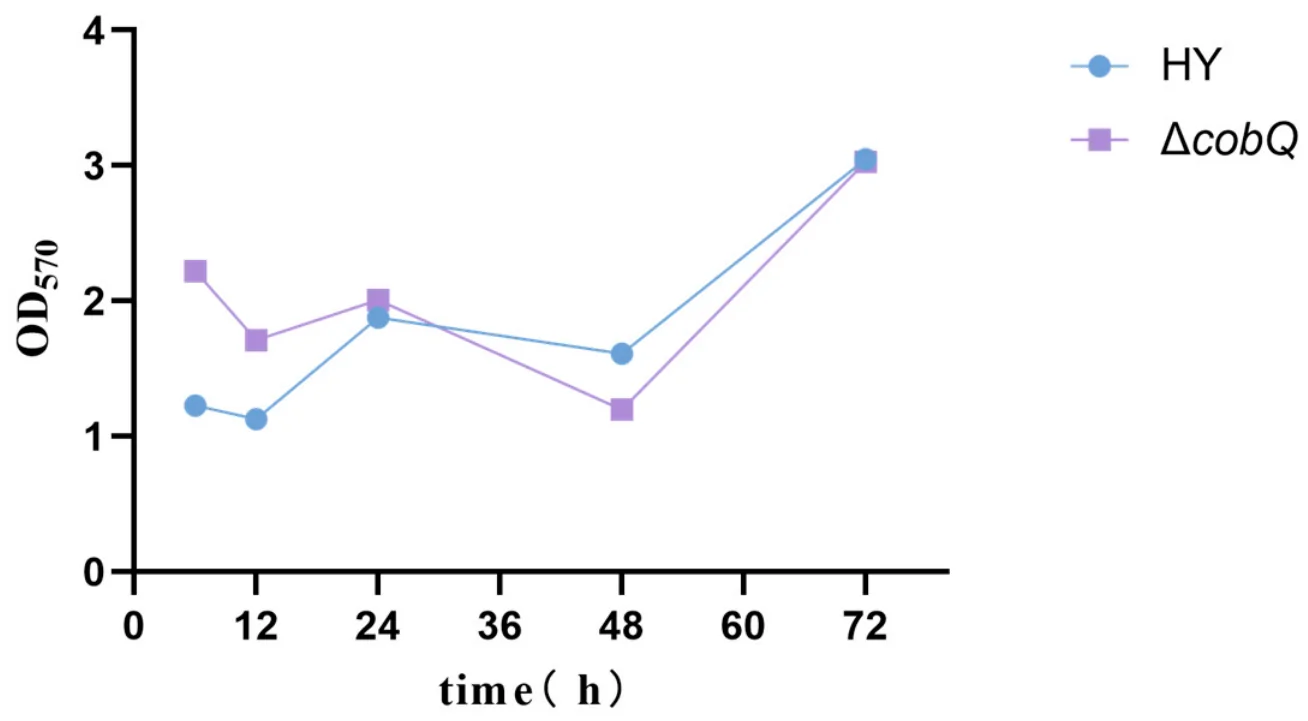
Measurement of biofilm formation.

Biofilm formation assay revealed that at early time points (6–12 h), the Δ*cobQ* strain exhibited enhanced biofilm formation compared to the wild-type strain (Figure 13). However, these results are based on technical replicates and should be considered descriptive observations only. The plasmid-complemented strain (C-*cobQ*) did not fully restore the wild-type phenotype and its results are shown in Supplementary Figure S2.

### 3.11. Effect of cobQ Deletion on Extracellular Protease Activity

**Figure 14:**
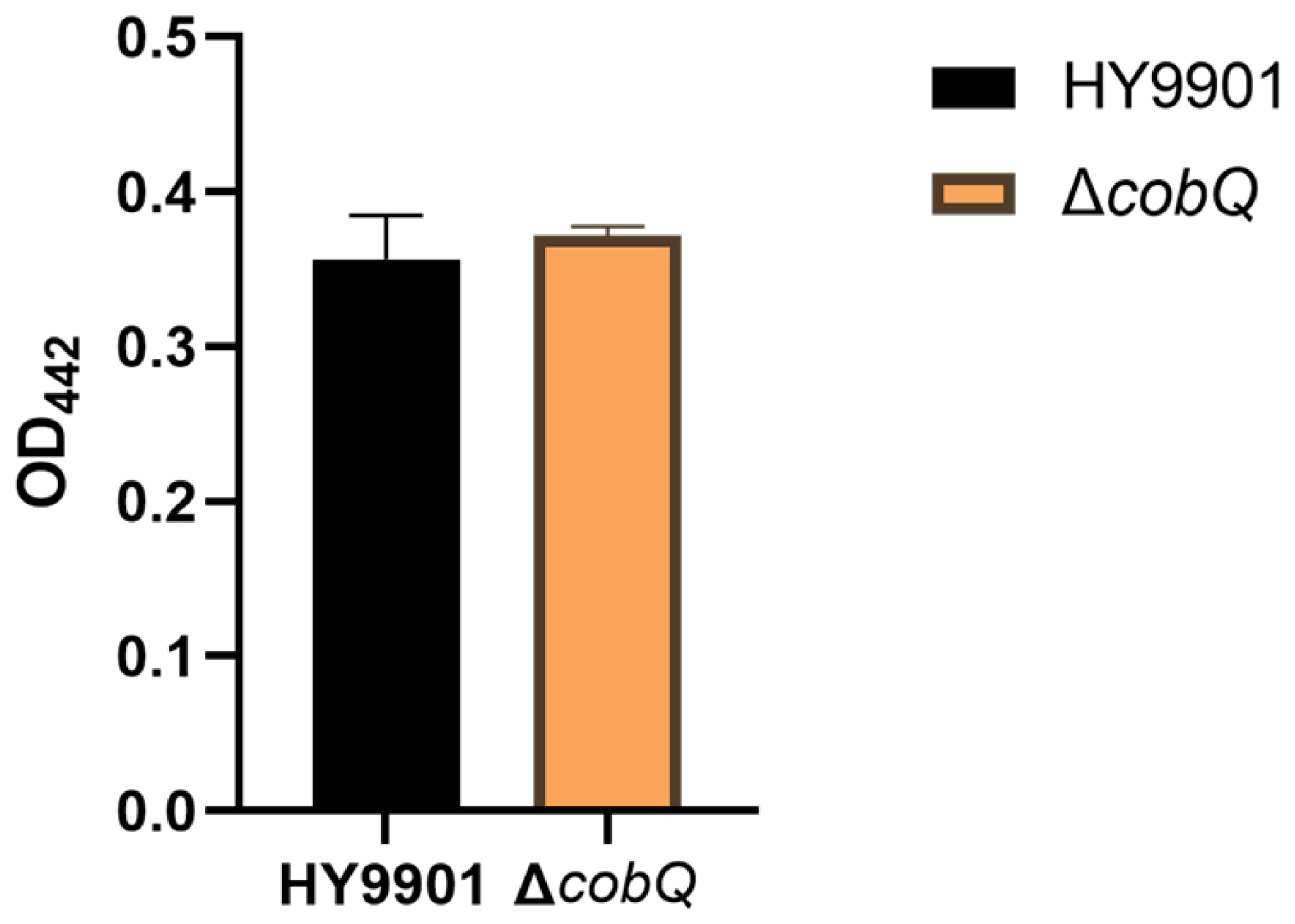
Extracellular protease activity.

Extracellular protease activity assay showed no significant difference between the wild-type strain HY9901 and the Δ*cobQ* mutant (*p* > 0.05), suggesting that *cobQ* deletion does not affect extracellular protease activity under the tested conditions. Based on the wild-type versus Δ*cobQ* comparison, we suggest that *cobQ* may not directly participate in the core regulatory pathway of extracellular protease activity under the tested conditions (Figure 14). The plasmid-complemented strain (C-*cobQ*) did not fully restore the wild-type phenotype and its results are shown in Supplementary Figure S3.

### 3.12. Effect of cobQ Deletion on Swimming Motility

Swimming motility assay showed that the Δ*cobQ* strain exhibited significantly reduced swimming motility at 0.3% agar (*p* = 0.0021) compared to the wild-type strain. No significant differences were observed at 0.4% agar (*p* = 0.2296) or 0.5% agar (*p* = 0.9201) (Table 4). The plasmid-complemented strain (C-*cobQ*) results are shown in Supplementary Table S1.

### 3.13. Effect of cobQ Deletion on Antimicrobial Susceptibility

Antimicrobial susceptibility testing revealed that compared to the wild-type HY9901, the Δ*cobQ* mutant showed decreased susceptibility to clindamycin, carbenicillin, and piperacillin, with inhibition zones reduced or absent. Conversely, the inhibition zone for cefuroxime increased, expanding from 15 mm to 29 mm and. These results suggest that *cobQ* deletion may affect antimicrobial susceptibility in *V. alginolyticus*. However, these results are based on technical replicates, the plasmid-complemented strain was not included, and species-specific CLSI breakpoints for *V. alginolyticus* have not been fully validated. Therefore, these results should be considered preliminary descriptive observations. The comprehensive results are presented in Table 5.

## 4. Discussion

Khib modification exhibits high conservation and functional importance in pathogenic bacteria. It was first systematically identified in *Proteus mirabilis*, where 1051 Khib-modified proteins were found to mainly participate in metabolic processes and stress responses [17]. In *Escherichia coli*, Khib modification is widespread on metabolic enzymes involved in glycolysis and the TCA cycle, and it also influences acid resistance by regulating the transcription factor H-NS [13]. In *Candida albicans*, Khib-modified proteins are enriched in ribosome biogenesis, carbon metabolism, and amino acid synthesis, suggesting a role in regulating protein translation [18]. In fungal pathogens such as *Ustilaginoidea virens* and *Fusarium graminearum*, Khib modification is directly involved in pathogenesis, and mutation of Khib sites on key virulence factors significantly reduces pathogenicity [19,20].

These prior studies demonstrate that Khib modification is widespread in pathogens and participates in regulating core physiological processes. The present study provides evidence that Khib modification is also widespread in *V. alginolyticus*, with 2848 Khib-modified proteins and 15,195 modification sites identified. The Co-IP experiment provided protein-level evidence for Khib on TpiA and Ndk (Figure 5), but does not constitute a strict orthogonal validation of the entire Khib dataset. Nevertheless, further orthogonal validation would be beneficial. The average density of more than five sites per protein is notably high, and false-positive identifications cannot be completely excluded. Independent MS/MS validation of a representative subset of sites would be required to further strengthen the confidence in individual assignments. Notably, 92% of the modified proteins identified in this study are involved in core biological processes, indicating that Khib participates broadly in physiological and metabolic regulation in *V. alginolyticus*. This comprehensive dataset provides a foundation for future elucidation of the Khib regulatory network in this aquatic pathogen.

To provide a more transparent assessment of site-level confidence, we re-analyzed the dataset under different localization probability and supporting peptide thresholds (Supplementary Table S2). Using a localization probability >0.90 and at least two supporting peptides, 5603 high-confidence sites were retained across 1827 proteins. When the threshold was raised to >0.99 with at least two supporting peptides, 5592 sites across 1819 proteins remained. This high-confidence subset provides a more conservative catalog of Khib sites and can be used for future targeted validation.

Research indicates that Khib modification targets key metabolic enzymes and regulates central carbon metabolism and energy conversion in both eukaryotes and prokaryotes [21]. In *P. mirabilis*, enolase (ENO) is modified by Khib at the K343 site, and molecular docking suggests this alters substrate binding conformation, thereby negatively regulating enzyme activity [17]. In *E. coli*, CobB regulates ENO activity by removing the K343 Khib modification, which in turn affects bacterial growth [12]. Our GO, KEGG, and COG analyses support these findings, showing that Khib-modified proteins are significantly enriched in metabolic pathways in *V. alginolyticus*. However, these bioinformatic analyses are descriptive in nature. While they reveal potential functional associations, they do not establish a causative relationship between Khib modification and specific biological functions. We therefore interpret these results as hypotheses-generating observations that require experimental validation.

Prior studies confirmed that Khib modification affects pathogenicity, but specific regulatory pathways were not well defined [19,22]. In the plant pathogen *U. virens*, Khib-modified proteins are enriched in pathogenesis pathways, and mutation of Khib sites reduces pathogenicity. Our study found that Khib-modified proteins were significantly enriched in pathways annotated as virulence-related, such as signal transduction and cell membrane biogenesis. This enrichment pattern suggests a potential role for Khib modification in virulence-associated processes, although direct functional validation is still needed [23,24,25,26]. These predictions are based on annotation and STRING analysis, and no direct pathogenicity assays were performed. Therefore, the link between Khib modification and virulence remains speculative and requires experimental validation.

Therefore, the core findings of this study are based on the comparison between HY9901 and Δ*cobQ*. The plasmid-complemented strain did not restore the wild-type phenotype, and its data are therefore presented as descriptive observations only. Synergistic regulation exists among different acylation modifications, as the same lysine residue can be targeted by multiple modifications, and competitive occupation or synergistic crosstalk can fine-tune protein function [14]. In *E. coli*, CobB possesses both deacetylase and de-Khib activity [12]. Our comparative analysis showed that some proteins are targeted by multiple acylation modifications, suggesting that Khib may function within a complex PTM regulatory network.

Recent studies have shown that CobQ homologs in other bacteria possess lysine deacetylase activity, suggesting a potential regulatory role for CobQ in lysine acylation modifications [27]. The present study suggests that CobQ may be associated with Khib modification in *V. alginolyticus*. In vitro assays showed a non-significant trend toward lower Khib levels in the presence of CobQ; therefore, no conclusion regarding de-Khib activity or cofactor dependence can be drawn. For causal inference, it would be necessary to demonstrate the restoration of global Khib levels in the plasmid-complemented strain or to use low-copy or chromosomal complementation. Until then, the result should be interpreted only as an association.

To better understand the potential function of CobQ, it is useful to compare it with known deacylases. CobB, a sirtuin-type enzyme, catalyzes deacylation through a conserved NAD^+^-dependent mechanism involving ADP-ribosylation of the acyl-lysine substrate [12]. Zn^2+^-dependent HDACs employ a metal-assisted water activation mechanism [10]. Domain analysis of CobQ using Pfam revealed the presence of multiple functional domains, including CbiA, ParA/MipZ, and AAA_31. Notably, none of these domains correspond to the conserved catalytic domains found in known deacylases, such as the sirtuin domain of CobB or the Zn^2+^-binding domain of HDACs. However, since no significant de-Khib activity was observed in vitro, the catalytic mechanism of CobQ remains unknown. However, since no significant de-Khib activity was observed in vitro, the catalytic mechanism of CobQ remains unknown. Future studies, including crystallography and site-directed mutagenesis, will be required to determine whether CobQ possesses de-Khib activity and, if so, to define its catalytic mechanism and relationship to other deacylase families. Several limitations of this study should be acknowledged. First, neither the transcriptional levels of *cobQ* were confirmed by qPCR, nor were the protein levels verified by Western blot due to the lack of a specific antibody. The expression status of *cobQ* in the knockout and plasmid-complemented strains was not examined. In addition, the plasmid-complemented strain did not restore the wild-type phenotype, so polar effects, secondary mutations, or non-specific consequences of the construct cannot be excluded. Second, in the Co-IP experiments (Figure 5), pre-immune serum was used as a negative control, but Input controls were not included, which limits the interpretation of the Co-IP results. Third, the in vitro de-Khib assay used Khib-modified BSA as a simplified model. While a non-significant trend toward lower Khib levels was observed in the presence of CobQ, this does not demonstrate de-Khib activity, and no conclusion regarding cofactor dependence or catalytic mechanism can be drawn. Fourth, it remains unclear whether the observed changes in Khib levels after *cobQ* deletion reflect a direct enzymatic effect of CobQ or an indirect consequence of metabolic reorganization. Fifth, the present study did not identify specific protein targets of CobQ. Future studies combining CobQ pull-down, targeted proteomics, or site-specific mutagenesis will be necessary to identify its direct substrates. Sixth, the high abundance of Khib sites identified does not necessarily imply that all of these modifications have a biological function; some may be non-functional or neutral events. Seventh, the antimicrobial susceptibility changes could not be specifically attributed to *cobQ*, as the plasmid-complemented strain was not included and S/I/R classifications were not assessed due to the lack of validated species-specific breakpoints. These results are therefore presented as descriptive observations only. Finally, the use of the 40B database instead of the HY9901 genome is a core limitation of this catalog, as it may lead to the omission of strain-specific proteins, incorrect peptide matching, and the loss of polymorphic sequences. A re-analysis against the HY9901 proteome was not performed in this study. The HY9901 genome is now available (PRJNA717128).

In summary, this study provides a comprehensive catalog of putative Khib-modified proteins in *V. alginolyticus* and reveals an increase in global Khib signal upon *cobQ* deletion. Our findings suggest that CobQ may be associated with Khib modification. However, the direct enzymatic function of CobQ, its specific substrates, the restoration of the phenotype in the plasmid-complemented strain, and the causal relationship with biofilm or antibiotic susceptibility have not been established. These findings provide a foundation for understanding the role of Khib modification in *V. alginolyticus* and identify candidate targets for future mechanistic studies.

## Figures and Tables

**Table 1:** Bacterial strains used in this study.

Strain	Description	Reference
*V. alginolyticus* HY9901	Wild-type strain	Laboratory stock
*E. coli* DH5α	Host for plasmid propagation	TransGen Biotech, Beijing, China
*E. coli* S17-1	Donor strain for conjugation	Laboratory stock
*E. coli* BL21(DE3)	Host for protein expression	TransGen Biotech, Beijing, China

**Table 2:** Plasmids used in this study.

Strain	Description	Reference
pMD18-T	TA cloning vector	Takara, Beijing, China
pLP12	Suicide plasmid for homologous recombination	Laboratory stock
pBBR1-MCS	Broad-host-range expression vector	Laboratory stock
pET-28a(+)	Expression vector for His-tagged protein	Laboratory stock

**Table 3:** Primers used in this study.

Primer	Sequence (5′→3′)	Reference
*cobQ*-F1	ATGATTGTTTGGAGTGTAGC	This study
*cobQ*-R1	TTACTGCTCATCGAACGC	This study
*cobQ*-MF1	GTGGCCCCGTTGCTCTACAAAAAAT	This study
*cobQ*-MR1	GATGAATCCTAAGCTAGGCCAACTT	This study
*cobQ*-MF2	TGATGTGCTGTCCAGTGAACAAGCA	This study
*cobQ* -MR2	AGTATCGACGTTCGATTCTATATTG	This study
Δ*cobQ*-yz-F	GTGGCCCCGTTGCTCTACAAAAAAT	This study
Δ*cobQ*-yz-R	AGTATCGACGTTCGATTCTATATTG	This study
Δ*cobQ*-F	GAACTCGAAACACAAGCAGATT	This study
Δ*cobQ*-R	CTGTTGATGTGCCAATTGCC	This study
C-*cobQ*-F	GGGTAAAAC CACCTCAACAGT	This study
C-*cobQ*-R	GGCTCTGGCTCTGGCTCT	This study
pLP12F	TACAGACAAACCAGAATCGG	This study
T3	ATTAACCCTCACTAAAGGGA	This study
pBBR1-YZ-F	ATGTGCTGCAAGGCGATTAAG	This study
pBBR1-YZ-R	TGACCATGATTACGCCAAGCG	This study

**Table 4:** Swimming motility.

Agar Concentration	HY9901	Δ*cobQ*	Significance
0.3%	30.2 ± 0.7	22.3 ± 0.5	*
0.4%	32.3 ± 1.3	28.5 ± 0.8	ns
0.5%	30.7 ± 0.5	32.0 ± 0.5	ns

Note: * *p* < 0.05 (Tukey-adjusted); ns, not significant.

**Table 5:** Drug sensitivity test results.

Drug	Dose (μg/disk)	Inhibition Zone Diameter (mm)	
		HY9901	Δ*cobQ*
Cefoperazone	75	18 ± 0.2	11 ± 0.3
Clindamycin	2	14 ± 0.3	7 ± 0.3
Penicillin	10 U	—	—
Ampicillin	100	—	—
Carbenicillin	100	6 ± 0.3	—
Cefazolin	30	11 ± 0.2	14.5 ± 0.3
Piperacillin	100	20 ± 0.1	—
Cefuroxime	30	15 ± 0.3	29 ± 0.1
Trimethoprim	23.75/1.25	21	21
Midecamycin	30	14	14
Vancomycin	30	11 ± 0.5	10
Polymyxin B	200 IU	15 ± 0.5	11 ± 0.2
Norfloxacin	10	20 ± 0.3	20.5 ± 0.2
Ofloxacin	5	20 ± 0.2	23.5 ± 0.4
Amikacin	30	17.5 ± 0.2	18 ± 0.2
Minocycline	30	21.1 ± 0.3	21.1 ± 0.1
Tetracycline	30	19.5 ± 0.1	20 ± 0.3
Gentamicin	10	19 ± 0.4	13.0 ± 0.2
Kanamycin	30	18 ± 0.1	0
Doxycycline	30	20 ± 0.1	21.2 ± 0.2

Note: The unit of inhibition zone diameter is mm; “—” indicates no inhibition zone or an indistinct inhibition zone. S/I/R classifications were not assessed. Statistical significance was not evaluated. The plasmid-complemented strain C-*cobQ* was not tested in this assay.

## Data Availability

The manuscript contains all of the data produced during this research. The processed LC-MS/MS search results, modified peptide table, and site localization scores generated in this study have been deposited to Figshare with the DOI: 10.6084/m9.figshare.33166328. The data are publicly available at: https://figshare.com/s/93ea3a8c2c5d0d47bc8b (accessed on 1 October 2026).

## Supplementary Materials

The following supporting information can be downloaded at: https://www.mdpi.com/article/10.3390/life16101698/s1, Figure S1: Growth curves of wild-type, Δ*cobQ*, and complemented strain C-*cobQ*; Figure S2: Biofilm formation of wild-type, Δ*cobQ*, and complemented strain C-*cobQ*; Figure S3: Extracellular protease activity of wild-type, Δ*cobQ*, and complemented strain C-*cobQ*; Table S1: Swimming motility of wild-type, Δ*cobQ*, and complemented strain C-*cobQ*. Table S2. Stepwise filtering strategy for the identification of high-confidence Khib sites.
