# Supplementary material for: Proteome-Wide Profiling of Lysine 2-Hydroxyisobutyrylation in Vibrio alginolyticus and Preliminary Association of CobQ with Global Khib Levels
Source: Life (Basel). 2026 Oct 10;16(10):1698. doi: 10.3390/life16101698 (PMC13706536; doi:10.3390/life16101698)
Supplement: Supplementary file 1 [file life-16-01698-s001.zip › life-4522524-supplementary.pdf]

Supplementary Materials

Proteome-Wide Profiling of Lysine 2-Hydroxyisobutyrylation in Vibrio alginolyticus and Functional Assessment of CobQ as a Candidate Khib Regulator

XiaoXin Wen, Shuai Yang, Yashu Liang, Yujia Zhang, Yingzhu Wei, Weijie Zhang, Jichang Jian, Na Wang, Bijian Pang, Huanying Pang *

This document contains supplementary figures and tables for the manuscript life-4444897.

Figure S1. Growth curves of wild-type, Δ*cobQ*, and complemented strain C-*cobQ*.

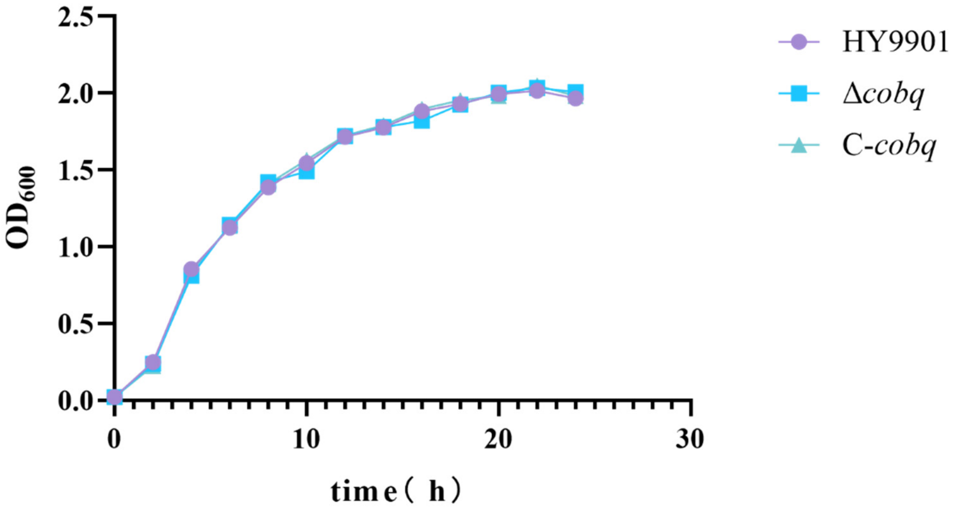

Growth curves of HY9901 (wild-type), Δ*cobQ*, and C-*cobQ* in TSB medium at 28 °C. OD_{600} values were measured at 2 h intervals for 24 h. No significant differences were observed among the three strains ($p > 0.05$, two-way ANOVA). Data are presented as mean ± SD (n = 3).

Figure S2. Biofilm formation of wild-type, Δ*cobQ*, and complemented strain C-*cobQ*.

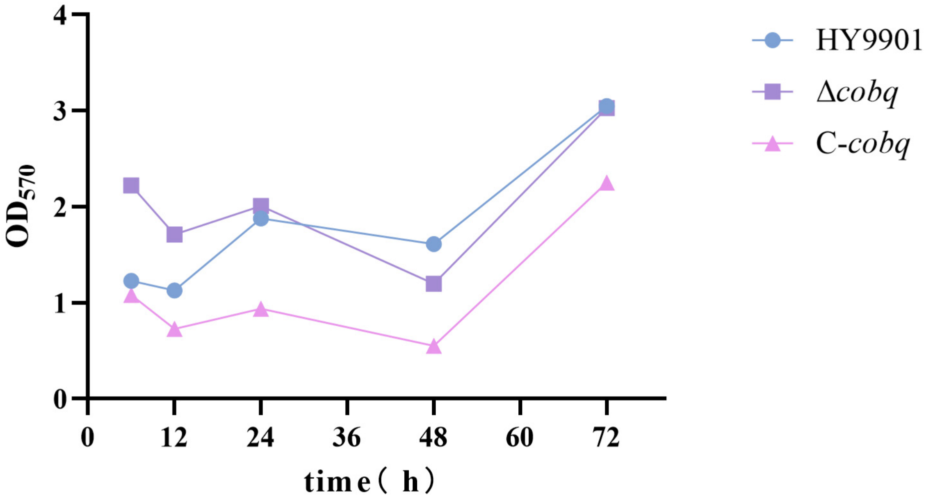

Biofilm formation of HY9901 (wild-type), Δ*cobQ*, and C-*cobQ* at different time points (6, 12, 24, 48, 72 h). The complemented strain showed lower biofilm formation than the wild-type at all time points, indicating that the complementation did not fully restore the wild-type phenotype. Data are presented as mean ± SD (n = 3).

Figure S3. Extracellular protease activity of wild-type, Δ*cobQ*, and complemented strain C-*cobQ*.

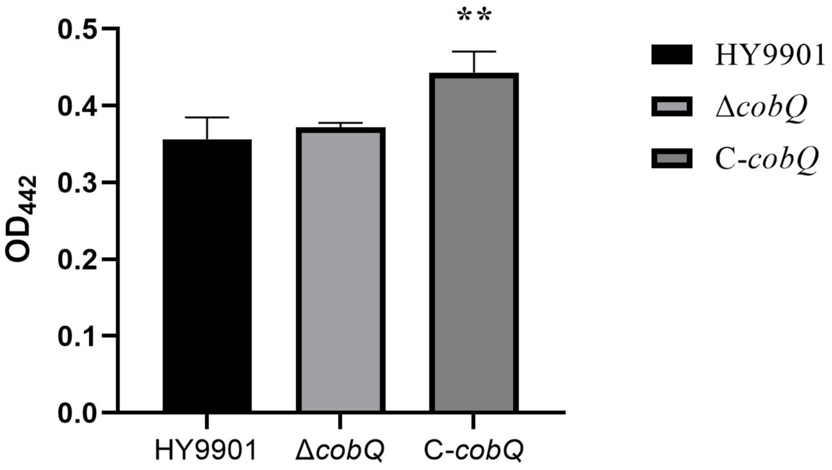

Extracellular protease activity of HY9901 (wild-type), Δ*cobQ*, and C-*cobQ*. The complemented strain exhibited significantly elevated protease activity compared to both wild-type and ΔcobQ strains ($p < 0.01$). Data are presented as mean ± SD (n = 3).

Table S1. Swimming motility of wild-type, Δ*cobQ*, and complemented strain C-*cobQ*.

Agar concentration	HY9901	Δ*cobQ*	*C-cobQ*
0.3%	30.2 ± 0.72	22.3 ± 0.45	26.3 ± 0.75
0.4%	32.3 ± 1.26	28.5 ± 0.77	31.4 ± 0.60
0.5%	30.7 ± 0.48	32.0 ± 0.51	30.0 ± 0.71

Note: Swimming diameter is measured in mm

Table S2. Stepwise filtering strategy for the identification of high-confidence Khib sites.

Localization probability	Supporting peptides	Retained sites	Retained proteins
> 0.90	⩾ 1	16,861	2848
> 0.95	⩾ 1	16,828	2848
> 0.90	⩾ 2	5,603	1827
> 0.95	⩾ 2	5,592	1822
> 0.99	⩾ 2	5,592	1819

Note: All filtering strategies were performed under strict criteria of single modification (Multiplicity = 1) and Protein FDR < 0.01. The ≥1 supporting peptide dataset represents the permissive catalog, whereas the ≥2 supporting peptides dataset represents the high-confidence catalog used for downstream interpretation.
